# CEBPB-mediated upregulation of SERPINA1 promotes colorectal cancer progression by enhancing STAT3 signaling

**DOI:** 10.1038/s41420-024-01990-9

**Published:** 2024-05-06

**Authors:** Yiming Ma, Ying Chen, Lei Zhan, Qian Dong, Yuanhe Wang, Xiaoyan Li, Lian He, Jingdong Zhang

**Affiliations:** 1 Department of Medical Oncology, Liaoning Cancer Hospital & Institute, Shenyang, Liaoning Province China; 2 Liaoning Key Laboratory of Gastrointestinal Cancer Translational Research, Shenyang, Liaoning Province China; 3 Department of Medical Oncology, The First Hospital of China Medical University, Shenyang, Liaoning Province China; 4 Department of Pathology, Liaoning Cancer Hospital & Institute, Shenyang, Liaoning Province China

**Keywords:** Oncogenes, Prognostic markers

## Abstract

Colorectal cancer (CRC) is a highly malignant carcinoma associated with poor prognosis, and metastasis is one of the most common causes of death in CRC. Serpin Family A Member 1 (SERPINA1) is a serine protease inhibitor from the Serpin family. Till now, the function and mechanism of SERPINA1 in CRC progression have not been fully illustrated. We established highly metastatic colorectal cancer cells named as RKO-H and Caco2-H by mice liver metastasis model. By integrative bioinformatic approaches, we analyzed the prognostic value and clinical significance of SERPINA1 in CRC, and predicted potential transcription factors. Colony formation, EDU, MTS, Transwell and wound healing assay were performed to evaluate the biological functions of SERPINA1 in CRC in vitro. Experiments in vivo were conducted to explore the effects of SERPINA1 on liver metastasis of CRC. ChIP and luciferase reporter gene assays were performed to identify the transcriptional regulatory mechanism of *SERPINA1* by CEBPB. Our results show that SERPINA1 is highly expressed in CRC and correlated with poor clinical outcomes. SERPINA1 promotes the proliferation, migration by activating STAT3 pathway. Mechanistically, CEBPB binds *SERPINA1* gene promoter sequence and promotes the transcription of *SERPINA1*. SERPINA1 drives CEBPB-induced tumor cell growth and migration via augmenting STAT3 signaling. Our results suggest that SERPINA1 is a potential prognostic marker and may serve as a novel treatment target for CRC.

## Introduction

Colorectal cancer is one of the most commonly diagnosed cancers affecting the digestive tract globally. Currently, its incidence rate is still increasing [1, 2]. In colorectal cancer, colon cancer accounts for about 60% and rectal cancer accounts for about 40%. The progression of colorectal cancer involves multiple stages, generally from normal mucosa to adenoma, and progresses finally to carcinoma [3, 4]. Due to the lack of early symptoms and specific biomarkers, many patients with colorectal cancer are diagnosed at later stages [5, 6]. Studies have shown that the development of colon cancer is related to diet and genetic factors, but its specific causes are still unclear [7, 8].

Approximately 20% of colon cancer patients have already developed to the metastatic state at the time of diagnosis, and it has been reported that over 30% of early-stage colon cancer patients will eventually develop into metastatic disease [9]. Once colon cancer cells undergo distant metastasis, treatment becomes palliative instead of curative. Liver is the most common site of CRC metastasis [10]. During the metastatic process, cancer cells exhibit certain features, including increased of cell adhesive factors and chemokine receptors, as well as changes in cell skeleton, which facilitate responses to chemotactic signals from distant organs and subsequent migration [11, 12]. In addition, the 5-year overall survival (OS) rate of metastatic CRC patients with liver resection for is only 47–60%, which is still unsatisfactory [13]. Therefore, exploring the molecular mechanisms related to the proliferation and metastasis of CRC is necessary.

Serpin Family A Member 1 (*SERPINA1*) encodes alpha-1-antitrypsin (A1AT) protein, according to the Gene Ontology (GO) database, this protein has functions such as protein binding, Golgi membrane binding, and protease binding [14]. Early studies of the *SERPINA1* gene mainly focused on A1AT deficiency, and related diseases including chronic liver disease, emphysema, and cardiovascular risk [15–18]. In recent years, the regulatory function of SERPINA1 in inflammation and immune responses has received more attention. Studies have shown that A1AT protein encoded by *SERPINA* can inhibit immune cells from releasing pro-inflammatory cytokines [19]. For the moment, our understanding of SERPINA1 in tumors is relatively limited. In non-small cell lung cancer, high expression of A1AT in plasma suggests that it is related to the poor prognosis of patients, and increased SERPINA1 in cell experiments has been shown to promote the proliferation and migration of lung cancer cells and inhibit apoptosis [20]. Previous study found that SERPINA1 was related to the early diagnosis of colorectal cancer. The tissue chip results show that by evaluating the combined results of SERPINA1, 96.77% of colorectal cancer tissues can be distinguished from normal tissues [21]. Previous studies have reported that A1AT and Snail can promote CRC metastasis and imply poor prognosis [22].

In the present study, we established highly metastatic colorectal cancer cells named as RKO-H and Caco2-H (H for highly metastatic). In vitro experiments, we verified their stronger proliferation and migration abilities compared to parental RKO and Caco2 cell lines. With high-throughput sequencing and public datasets, we analyzed and screened SERPINA1 as the potential oncogenic gene, which leading to progression of CRC. Through experimental verification, we found that SERPINA1 can promote the proliferation, migration by activating STAT3 pathway. Our research provided a novel perspective on the mechanisms underlying CRC.

## Results

### RKO-H and Caco2-H have higher proliferation and migration capabilities than parental RKO and Caco2

The schematic diagram (Supplementary Fig. 1A) shows the establishment process of RKO-H and Caco2-H cell lines. RKO and Caco2 cells were injected into nude mice by splenic injection, and CRC from the liver metastasis lesion were isolated for primary culture, which was repeated four times to obtain separated and cultured cells defined as RKO-H and Caco2-H. Colony formation assay showed that the number of colonies formed by RKO-H was significantly increased compared to RKO (*P* < 0.001) (Supplementary Fig. 1B), and the same was observed for Caco2-H compared to Caco2 (*P* < 0.001) (Supplementary Fig. 1C). Transwell migration assays revealed a significant increase in the number of RKO-H cells passing through the chamber compared to RKO (*P* < 0.01) (Supplementary Fig. 1D), as well as for Caco2-H compared to Caco2 (*P* < 0.001) (Supplementary Fig. 1E). Wound healing assays showed a significant increase in the healing area of RKO-H compared to RKO (*P* < 0.01) (Supplementary Fig. 1F), and a significant increase in the proportion of healing area of Caco2-H compared to Caco2 (*P* < 0.05) (Supplementary Fig. 1G). These results indicated that RKO-H and Caco2-H had enhanced proliferation and migration abilities compared with RKO and Caco2.

### Identification of SERPINA1 as a potential factor in CRC metastasis

Based on the above results, we found that RKO-H had higher proliferation and migration abilities than parental RKO cells, and so was as Caco2-H compared to parental Caco2 cells. To further explore the molecular and mechanistic changes underlying the phenomena, we analyzed the RNA sequencing results of these cell groups. We obtained 1224 differentially expressed genes in RKO-H compared to RKO, including 842 up-regulated genes and 382 down-regulated genes (Supplementary Fig. 2A). We also obtained 880 differentially expressed genes in Caco2-H compared to Caco2, including 411 up-regulated genes and 469 down-regulated genes (Supplementary Fig. 2B). The heatmap depicts the top 50 differentially expressed genes with the largest |log2FC| between RKO-H and RKO cell groups, which include genes such as SERPINA1, NUPR1, GAP43, MSN, FMN1 and PSD3 (Supplementary Fig. 2C); the heatmap also depicts the top 50 differentially expressed genes with the largest |log2FC| between Caco2-H and Caco2 cell groups, which include CACNA2D1, SEMA3D, TNFSF8, STAP2, TUBAL3 and PAPSS2. To obtain genes related to colorectal cancer liver metastasis, we analyzed the colorectal cancer liver metastasis dataset GSE49355. The data analysis results showed that there were 257 differentially expressed genes between liver metastases and primary tumors, including 160 up-regulated genes and 97 down-regulated genes in liver metastases (Supplementary Fig. 2D). The Venn diagram shows that SERPINA1 is the only intersection the top 50 differentially expressed genes between GSE49355 and RKO-H/RKO cell groups as well as between Caco2-H/Caco2 cell groups (Supplementary Fig. 2E), suggesting that SERPINA1 plays an important role in colorectal cancer liver metastasis.

### Expression levels of SERPINA1 in CRC

**Fig. 1:**
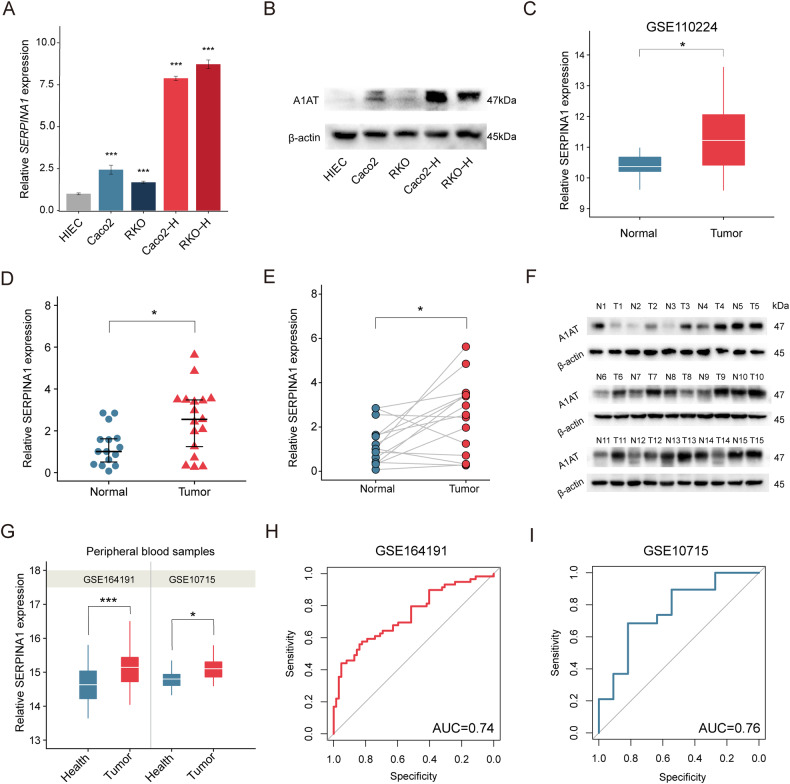
Expression levels of SERPINA1 in CRC. **A** RT-qPCR detection of SERPINA1 mRNA levels in HIEC, RKO, Caco2, RKO-H, and Caco2-H cells. Error bars: standard deviations. **B** Western blot detection of A1AT protein levels in HIEC, RKO, Caco2, RKO-H and Caco2-H cells. **C** Box plot showing the expression levels of CRC SERPINA1 in GSE110224 (*n* = 34). **D** RT-qPCR detection of CRC SERPINA1. **E**, **F** RT-qPCR and western detection of SERPINA1 levels in 15 pairs of CRC and adjacent tissues. N refers to normal and T refers to tumor. **G** Box plot showing the expression levels of SERPINA1 in the blood of colorectal cancer patients and healthy individuals. **H** ROC curve for blood SERPINA1 levels based on GSE164191 (*n* = 121). **I** ROC curve for blood SERPINA1 levels based on GSE10715 (*n* = 30). *P < 0.05, **P < 0.01, ***P < 0.001.

The RT-qPCR results showed that SERPINA1 expression in RKO, Caco2, RKO-H, and Caco2-H cells were higher than that in HIEC (*P* < 0.001) (Fig. 1A), and the SERPINA1 expression in RKO-H and Caco2-H cells was higher than that in RKO and Caco2 cells, respectively. Western blot further showed the increased protein expression of A1AT in RKO, Caco2, RKO-H, and Caco2-H cells compared to HIEC cells, with the highest expression observed in RKO-H and Caco2-H cells (Fig. 1B). In the GSE110224 dataset, the expression level of SERPINA1 in CRC was significantly higher than that in control tissues (*P* < 0.05) (Fig. 1C). We also validated these findings in fresh tissue samples, showing that the mRNA expression of SERPINA1 was significantly higher in CRC samples than in control samples (*P* < 0.05) (Fig. 1D), and paired test also showed similar results (*P* < 0.05) (Fig. 1E). Western blot analysis further confirmed the increased protein level of A1AT in CRC tissues compared to the paired control tissues (Fig. 1F). Additionally, GSE164191 and GSE10715 datasets showed that the peripheral blood mRNA levels of SERPINA1 in colorectal cancer patients was higher than that in healthy population (Fig. 1G). Receiver operating characteristic (ROC) curve showed diagnostic accuracy of peripheral blood SERPINA1 expression in discriminating colorectal cancer patients from healthy population, with area under the curve (AUC) of 0.74 and 0.76, respectively (Fig. 1H, I). In summary, our findings based on public datasets, cell lines and tissues demonstrated that A1AT is upregulated in colorectal cancer compared to normal tissues.

### Prognostic analysis of SERPINA1 in CRC

**Fig. 2:**
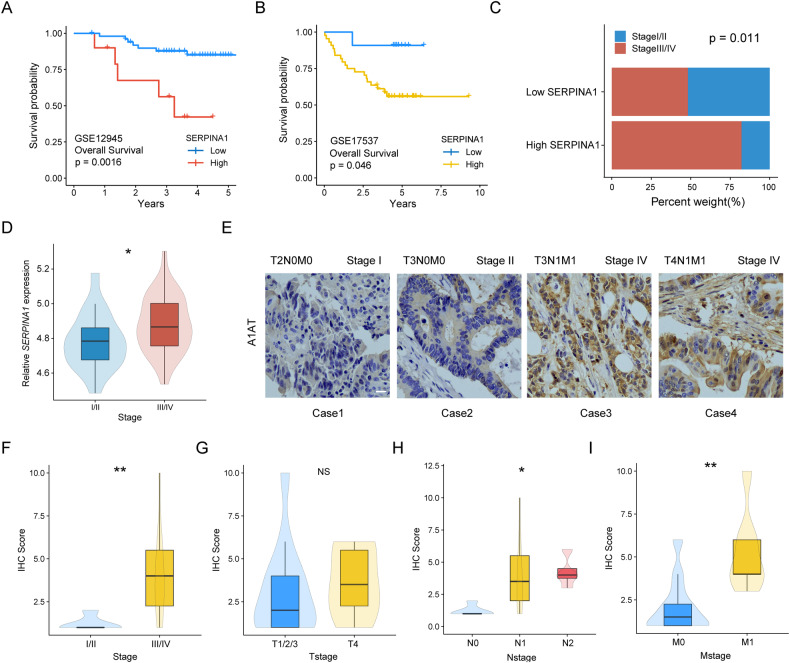
Prognostic and TNM stage analysis of SERPINA1. **A** Based on the GSE12945 dataset, the KM curve shows the relationship between SERPINA1 and OS (*n* = 62). **B** Based on the GSE17537 dataset, the KM curve shows the relationship between SERPINA1 and OS (*n* = 55). **C** The proportion of Stage III/IV patients in the high expression group of SERPINA1 is significantly higher than that in the low expression group. **D** The expression of A1AT in different stages of CRC. **E** Immunohistochemical staining images of A1AT in colorectal cancer patients of T2N0M0, T3N0M0, T3N1M1 and T4N1M1. Scale bar: 50 μm. **F**–**I** IHC results show that A1AT expression is related to advanced pathological stage, N stage and M stage, but not associated with advanced T stage. NS : *P* ≥ 0.05, *P < 0.05, **P < 0.01.

Kaplan-Meier curves were plotted with SERPINA1 to demonstrate the relationship between SERPINA1 expression and OS. CRC patients with higher SERPINA1 had worse OS than those with lower SERPINA1 expression (*P* = 0.0016, *P* = 0.046) (Fig. 2A, B). In GSE17537, patients were divided into two groups according to the median SERPINA1 expression level. The distribution of TNM stages was analyzed, with 82% of Stage III/IV patients having high SERPINA1 expression compared to 48% of Stage III/IV patients in the low SERPINA1 expression group. Fisher’s exact test showed that there was a significant difference in TNM stage between the high and low SERPINA1 expression groups (*P* = 0.011) (Fig. 2C).

Furthermore, the expression of SERPINA1 was significantly higher in CRC patients with advanced stages (*P* < 0.025) (Fig. 2D). These findings suggest that SERPINA1 may be associated with poor prognosis and clinical stage progression of colorectal cancer. This was subsequently validated using immunohistochemistry (IHC). IHC showed representative IHC staining images of A1AT for different TNM stages (Fig. 2E). The IHC results demonstrated that the expression of A1AT was in CRC patients with advanced stages (*P* < 0.01) (Fig. 2F), while there was no significant difference in A1AT expression between T4 and T1/2/3 stage patients (*P* > 0.05) (Fig. 2G). In addition, the expression of A1AT was higher in patients with advanced N stage (*P* < 0.05) (Fig. 2H) and M1 stage (*P* < 0.01) (Fig. 2I) than in patients with lower stages. These results found that A1AT might be associated with clinical progression and poor prognosis of CRC.

### SERPINA1 promotes the proliferation and migration of CRC cells in vitro

**Fig. 3:**
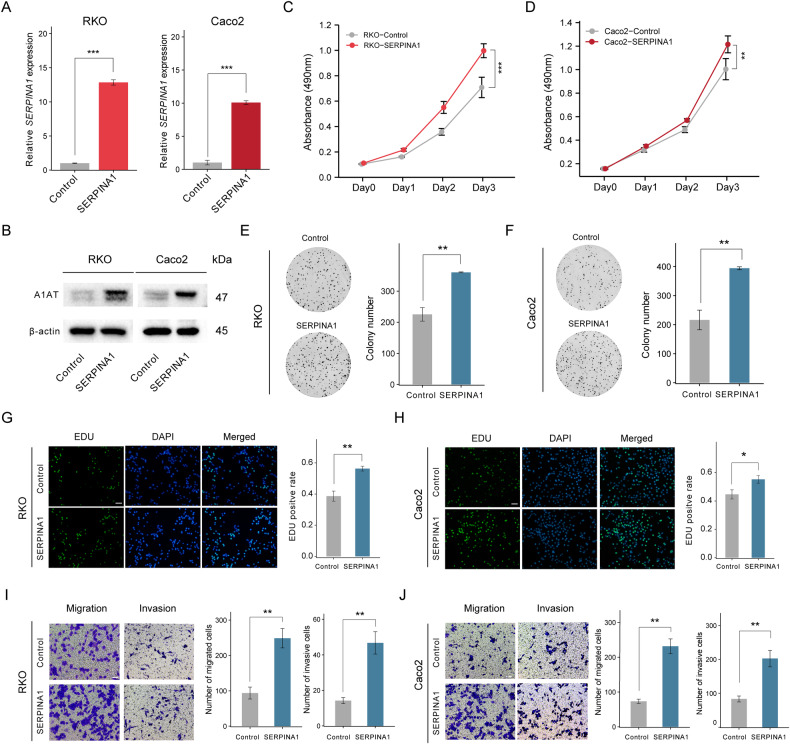
SERPINA1 promotes proliferation and migration abilities of colorectal cancer. **A**, **B** In RKO and Caco2 cell lines, SERPINA1 expression levels in Control group and the SERPINA1 overexpression group were verified by RT-qPCR and Western blot. **C**, **D** MTS showing absorbance values of RKO and Caco2 cells on day 0, day 1, day 2, and day 3. **E**, **F** Colony formation images of RKO and Caco2 cells. **G**, **H** EDU staining images of RKO and Caco2 cells in the Control group and SERPINA1 group. Scale bar: 50 μm. **I**, **J** Transwell migration and invasion result images of RKO cells and Caco2 cells. Scale bar: 50 μm. *P < 0.05, **P < 0.01, ***P < 0.001. Control: control group; SERPINA1: SERPINA1 overexpression group. Error bars: standard deviations.

First, we used SERPINA1 overexpression and control lentivirus to transfect RKO and Caco2 cell lines. The PCR and Western blot results showed that in RKO and Caco2 cell lines, the SERPINA1 overexpression group had a significant increase in SERPINA1 levels compared to the control group (*P* < 0.001) (Fig. 3A, B).MTS results showed that the absorbance value of RKO cells with SERPINA1 overexpression significantly increased on the day 3 compared to the control group (*P* < 0.001) (Fig. 3C), and the absorbance value of Caco2 cells with SERPINA1 overexpression group was significantly increased on day3 compared to the control group (*P* < 0.01) (Fig. 3D). SERPINA1 overexpression induced more colony numbers of RKO and Caco2 cells (*P* < 0.01) (Fig. 3E, F). Meanwhile, the EDU staining positivity rates of RKO and Caco2 cells were increased, which was induced by SERPINA1 overexpression (*P* < 0.05) (Fig. 3G, H). Transwell experiment results showed that SERPINA1 overexpression could promote the migration and invasion ability of colorectal cancer cells (*P* < 0.01) (Fig. 3I, J).

**Fig. 4:**
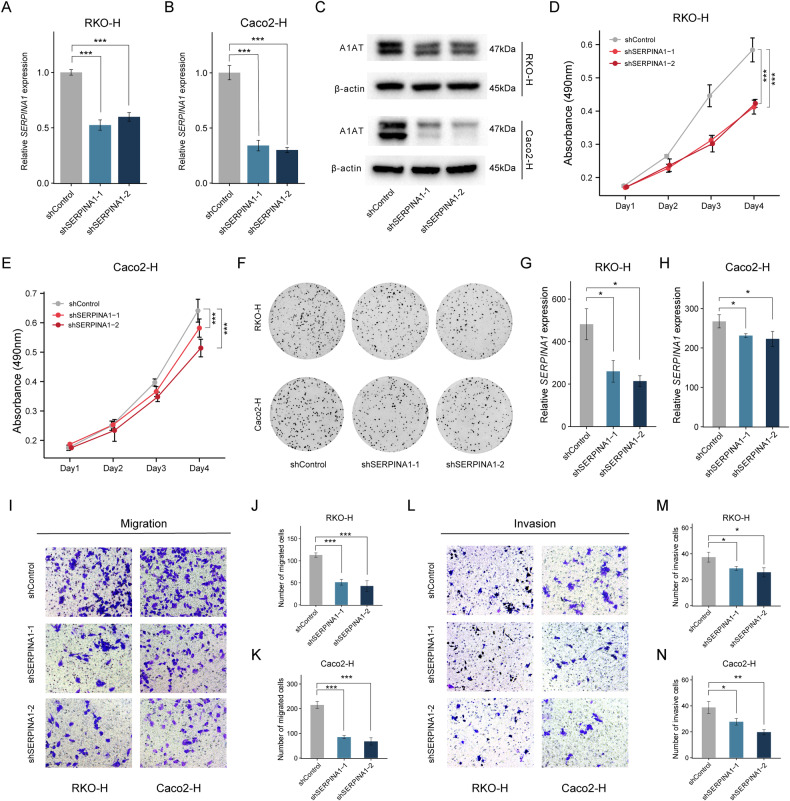
Reduced SERPINA1 expression inhibits the proliferation ability of CRC. **A**, **B** In RKO-H and Caco2-H cells, the mRNA expression of SERPINA1 in the shControl group, shSERPINA1-1 group, and shSERPINA1-2 group. **C** The A1AT expression in the shControl group, shSERPINA1-1 group, and shSERPINA1-2 group was verified by Western blot. **D** MTS shows the absorbance values of RKO-H cells in the shControl group, shSERPINA1-1 group and shSERPINA1-2 group on day 0, day 1, day 2, and day 3. **E** MTS shows the absorbance values of Caco2-H cells in the shControl group, shSERPINA1-1 group, and shSERPINA1-2 group on day 0, day 1, day 2, and day 3. **F**–**H** Colony formation images of RKO-H and Caco2-H cells in the shControl group, shSERPINA1-1 group, and shSERPINA1-2 group. **I**–**K** Transwell migration images of RKO-H and Caco2-H cells in the shControl group, shSERPINA1-1 group, and shSERPINA1-2 group. Scale bar: 50 μm. **L**–**N** Transwell invasion images of RKO-H and Caco2-H cells in the shControl group, shSERPINA1-1 group, and shSERPINA1-2 group. Scale bar: 50μm. *P < 0.05, **P < 0.01, ***P < 0.001. shControl: control group; shSERPINA1-1: SERPINA1knockdown group 1; shSERPINA1-2: SERPINA1knockdown group 2. Error bars: standard deviations.

Next, we established SERPINA1 knockdown cell lines using RKO-H and Caco2-H cells. PCR results showed SERPINA1 expression in the SERPINA1 Knockdown group were significantly reduced compared to the Control groups (Fig. 4A–C). The results of Colony formation and MTS showed that knockdown of SERPINA1 inhibited the proliferation of RKO-H and Caco2-H cells (Fig. 4D–H). Transwell migration and Transwell invasion experiments indicated that less tumor cells traversed the chambers in the SERPINA1 Knockdown group (Fig. 4I–N). These experimental results suggest that upregulation of SERPINA1 can enhance proliferation and migration abilities of CRC cells.

### SERPINA1 exerts pro-tumor effects via activating the STAT3 signaling pathway

**Fig. 5:**
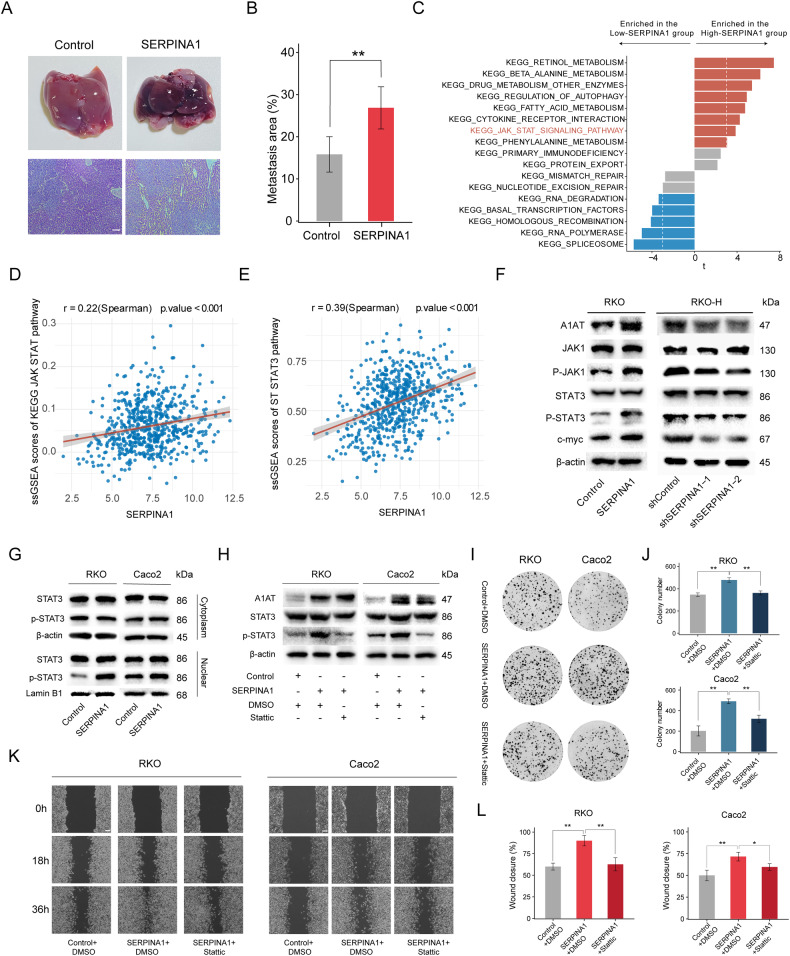
SERPINA1 positively regulates JAK1-STAT3 pathway to promote proliferation and migration of colorectal cancer. **A** Representative images of liver and corresponding HE staining images of RKO-Control and RKO-SERPINA1 splenic injection liver metastasis model (*n* = 10). Scale bar: 100 μm. **B** Bar graph comparing the proportion of liver metastasis area in the two groups of cells. **C** GSVA enrichment for SERPINA1. x-axis representing the t-value of limma analysis and the y-axis representing the KEGG pathway. **D**, **E** Correlation analyses of SERPINA1 and scores of STAT3 pathways (KEGG JAK STAT and ST STAT3). **F** Western blot of A1AT, p-STAT3, STAT3, p-JAK1 and JAK1 in RKO and RKO-H. **G** Western blot confirmed the effect of SERPINA1 overexpression on p-STAT3 nuclear entry in RKO and Caco2 cell lines. **H** Western blot analyzed the expression of A1AT, STAT3, p-STAT3, and β-actin in the control group, SERPINA1 overexpression group, and SERPINA1 overexpression plus p-STAT3 inhibitor group. **I**, **J** Colony formation assay of RKO and Caco2 cells showed that overexpression of SERPINA1 significantly increased colony formation, while addition of p-STAT3 inhibitor significantly reduced tumor colony formation. **K**, **L** Wound healing assay of RKO and Caco2 cells showed that overexpression of SERPINA1 significantly increased would healing area, while STAT3 inhibitor significantly reduced would healing area. Scale bar: 100 μm. *P < 0.05, **P < 0.01, ***P < 0.001. Error bars: standard deviations.

Further, the relationship between SERPINA1 and colorectal cancer liver metastasis was investigated through in vivo model. RKO-Control and RKO-SERPINA1 were injected into the spleens of five nude mice, respectively. Subsequently, HE (hematoxylin-eosin) staining was performed with the liver metastatic tumor (Fig. 5A). The proportion of liver metastasis area increased in SERPINA1 overexpression group (Fig. 5B).

Based on GSVA enrichment analysis, KEGG pathways of RETINOL METABOLISM, REGULATION OF AUTOPHAGY and JAK STAT were significantly enriched in the High-SERPINA1 group. KEGG pathways of RNA DEGRADATION, BASAL TRANSCRIPTION FACTORS and HOMOLOGOUS RECOMBINATION were significantly enriched in the Low-*SERPINA1* group (Fig. 5C). Based on the ssGSEA prediction results, SERPINA1 was positively correlated with the KEGG JAK STAT score (Fig. 5D) and the ST STAT3 score (Fig. 5E). Since the JAK STAT3 pathway is known to be involved in the proliferation and migration of CRC, we chose this pathway for validation. Western blot results showed that A1AT in RKO overexpression cells upregulated p-JAK1, p-STAT3, and c-MYC, while STAT3 and JAK1 showed no significant changes; knocking down SERPINA1 in RKO-H cells downregulated p-JAK1, p-STAT3, and c-MYC, while STAT3 and JAK1 showed no significant changes (Fig. 5F). Western blot results showed that in RKO and Caco2 cell lines, overexpression of SERPINA1 increased the nuclear distribution of p-STAT3, while there was no significant difference between the two groups in the cytoplasm (Fig. 5G).

Based on these results, we further examined whether A1AT promote CRC progression by STAT3 pathway. Western blot results showed that Stattic could inhibit p-STAT3 expression (Fig. 5H). Colony formation experiments showed that compared with the SERPINA1 + DMSO group, the SERPINA1+Stattic group showed a significant decrease in the number of colonies formed (Fig. 5I, J). Wound healing results showed that the SERPINA1+Stattic group showed a significant decrease of the proportion of healing area, compared with the SERPINA1 + DMSO group (Fig. 5K, L). These results indicate that SERPINA1 may induce proliferation and metastasis of CRC via activating STAT3 pathway.

### Transcription factors associated with *SERPINA1*

**Fig. 6:**
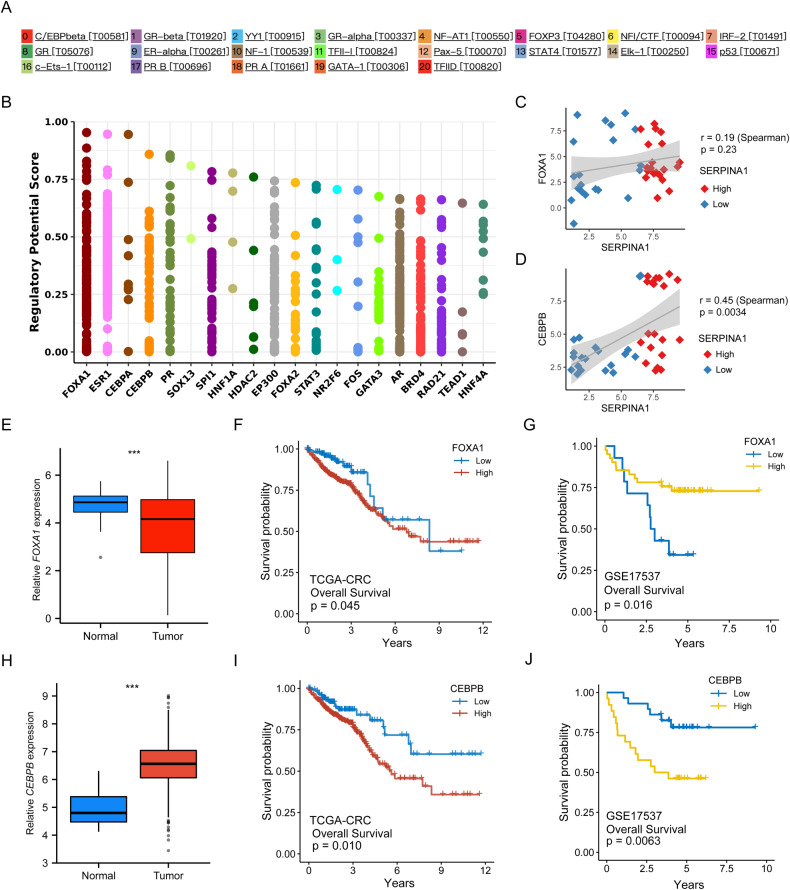
Prediction of transcription factors related to SERPINA1. **A** PROMO database predicts transcription factors related to SERPINA1. **B** Cistrome database predicts transcription factors related to SERPINA1. **C**, **D** Based on GSE19860, correlation analyses of SERPINA1 and transcription factors (FOXA1and CEBPB). **E** Expression levels of FOXA1 in tumor and control groups in TCGA-CRC dataset. **F** Relationship between FOXA1 expression and OS of TCGA-CRC patients (*n* = 612). **G** Relationship between FOXA1 expression and OS of CRC patients in GSE17537 (*n* = 55). **H** Expression levels of CEBPB in tumor and control groups in TCGA-CRC dataset. **I** Relationship between CEBPB expression and TCGA-CRC patients (*n* = 612). **J** Relationship between CEBPB expression and OS of CRC patients in GSE17537. ***P < 0.001. Error bars: standard deviations.

We obtained 20 transcription factors potentially related to SERPINA1, including CEBPB, GR-beta, YY1, GR-alpha, NF-AT1, FOXP3, etc, based on the PROMO database (Fig. 6A). The Cistrome database was also used to predict 20 transcription factors related to *SERPINA1*, including FOXA1, ESR1, CEBPA, CEBPB, PR, etc. (Fig. 6B).

We chose the top-ranked CEBPB and FOXA1 for further analyses. Based on the colorectal cancer dataset GSE19860, Spearman’s method was used to analyze the correlation between SERPINA1 and FOXA1 (*r* = 0.19, *P* = 0.23) (Fig. 6C) and the correlation between SERPINA1 and CEBPB (*r* = 0.45, *P* = 0.0034) (Fig. 6D). The results suggest that SERPINA1 and CEBPB are positively correlated in colorectal cancer. Here we used a public colorectal cancer dataset to analyze the prognostic significance and clinical relevance of FOXA1 and CEBPB. Based on the TCGA-CRC dataset, FOXA1 expression was significantly lower than that in adjacent non-tumor tissue (*P* < 0.001) (Fig. 6E), and KM curves showed that FOXA1 expression was not related to OS in TCGA colorectal cancer patients (*P* = 0.045) (Fig. 6F). KM curves showed that FOXA1 expression was related to OS in GSE17537 colorectal cancer patients but the curves crossed (*P* = 0.016) (Fig. 6G). Expression of FOXA1 was correlated with earlier pathological stages of colorectal cancer (*P* < 0.001), and was not correlated with T stage (*P* > 0.05), but was correlated with N stage (*P* < 0.001) and M stage (*P* < 0.001) (Supplementary Fig. 3A–D). Based on the TCGA-CRC dataset, CEBPB expression was significantly higher compared to adjacent non-tumor tissue (*P* < 0.001) (Fig. 6H), and KM curves showed that OS and disease-specific survival were shorter in the high CEBPB expression group (*P* = 0.010) (Fig. 6I). In the GSE17537 dataset, overall survival was shorter in the high CEBPB expression group of colorectal cancer patients (*P* = 0.0063) (Fig. 6J). Expression of CEBPB was correlated with more advanced pathological stages of colorectal cancer (*P* < 0.05), was not associated with T stages (*P* > 0.05), but was correlated with N stages (*P* < 0.05) and M stages (*P* < 0.01) (Supplementary Fig. 3E–H).

### CEBPB regulates *SERPINA1* transcription

**Fig. 7:**
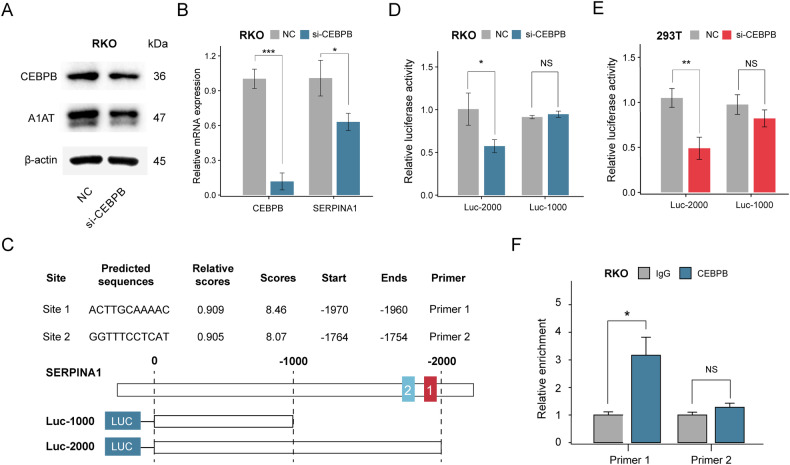
Transcription factor CEBPB positively regulates the transcription of SERPINA1. **A**, **B** Western blot and RT-qPCR confirmed the regulation of CEBPB on SERPINA1. **C** JASPAR database predicts potential binding sites of transcription factor CEBPB on SERPINA1 promoter. **D**, **E** Dual luciferase reporter gene assay confirmed the effect of CEBPB on fluorescence activity of transfected Luc-2000 and Luc-1000 in RKO and 293T cells. **F** ChIP-pcr using CEBPB antibody and IgG antibody in RKO cells to verify CEBPB-related binding sites. NS : *P* ≥ 0.05, *P < 0.05, **P < 0.01, ***P < 0.001. Error bars: standard deviations.

**Fig. 8:**
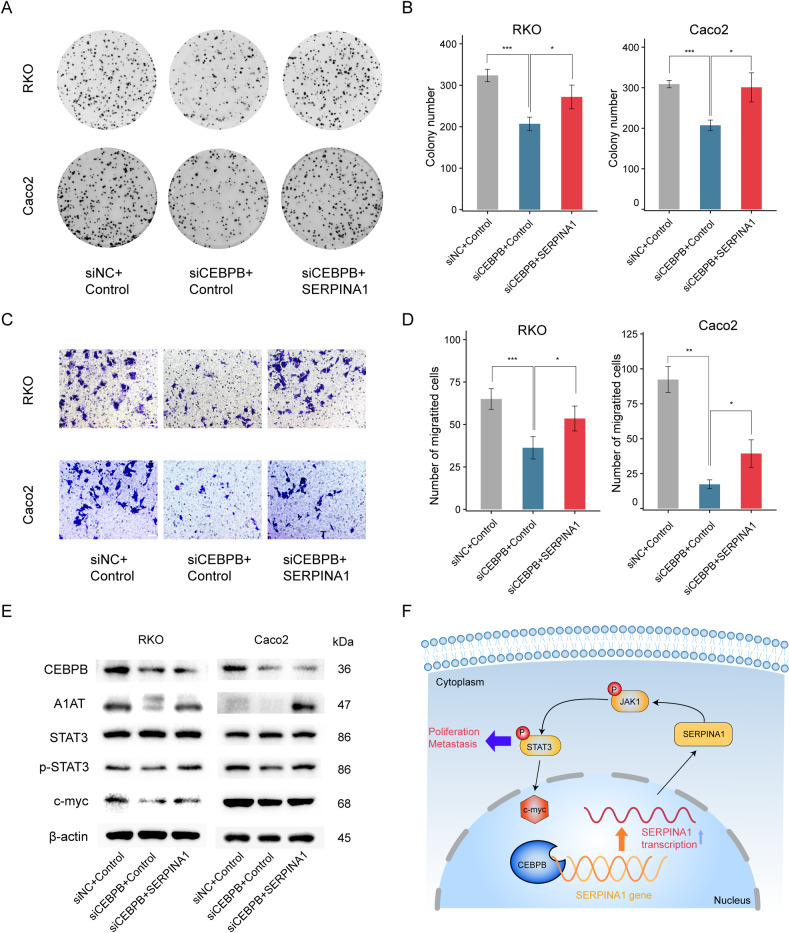
SERPINA1 mediates CEBPB-induced promotion of CRC via STAT3 signaling pathway. **A**, **B** Colony formation assays showed that increased SERPINA1 could restore the inhibition of colorectal cancer cell proliferation by knocking down CEBPB. **C**, **D** Transwell assays showed that increased SERPINA1 could restore the inhibition of colorectal cancer cell migration by knocking down CEBPB. **E** Western blot verification of protein expression of STAT3, p-STAT3 and c-myc after knocking down CEBPB and overexpressing SERPINA1. **F** Schematic diagram of our research. *P < 0.05, **P < 0.01, ***P < 0.001. Error bars: standard deviations.

Based on data from public datasets, the transcription factor CEBPB is more valuable than FOXA1 in evaluating the prognosis of colorectal cancer, so we further explored the regulatory relationship between CEBPB and SERPINA1. After knocking down CEBPB, the protein and mRNA levels of SERPINA1 were significantly reduced, indicating that CEBPB has a positive regulatory effect on SERPINA1 (Fig. 7A, B). The JASPAR database was used to predict binding sites related to the CEBPB transcription factor and the *SERPINA1* promoter sequence. Two potential binding sites (Relative scores > 0.9) were selected, located at -1970bp to −1960 bp and −1764 bp to −1754bp in the promoter region of the *SERPINA1* gene sequence. Both potential binding sites were located at -2000bp to −1000bp (Fig. 7C). Dual luciferase reporter gene assays confirmed that CEBPB has a transcriptional regulatory effect on *SERPINA1* in 293T and RKO cell lines. After knocking down CEBPB, the luciferase activity of the SERPINA1 promoter (Luc-2000) was significantly reduced (Fig. 7D, E), while knocking down CEBPB had no significant effect on the luciferase activity of the SERPINA1 promoter (Luc-1000). This indicates that CEBPB has a positive transcriptional regulatory effect on *SERPINA1* and that the potential binding site is located at −2000 bp to −1000 bp in the *SERPINA1* promoter sequence. ChIP experiments confirmed that the relative enrichment level of primer 1 in the CEBPB antibody group was significantly higher than that of primer 1 in the IgG antibody group (*P* < 0.05), while there was no significant difference between the relative enrichment level of primer 1 in the CEBPB antibody group and that of primer 2 in the IgG antibody group (*P* > 0.05) (Fig. 8F). These results indicate that CEBPB binds to site 1 of the *SERPINA1* gene promoter sequence and promotes the transcription of *SERPINA1*.

### SERPINA1 mediates CEBPB-induced promotion of CRC via STAT3 signaling pathway

To further verify the regulatory relationship between CEBPB and SERPINA1 and their effects on colorectal cancer proliferation, we transfected RKO and Caco2 cells with si-NC and Control, si-CEBPB and Control, and si-CEBPB and SERPINA1, respectively, and performed colony formation assays. The results of the RKO and Caco2 colony formation assays showed that the number of colonies formed in the si-CEBPB+Control group was significantly lower than that in the si-NC+Control group; the number of colonies formed in the si-CEBPB + SERPINA1 group was significantly higher than that in the si-CEBPB+Control group (Fig. 8A, B). Transwell migration assay results showed that the number of migrating cells in the si-CEBPB+Control group was significantly lower than that in the si-NC+Control group; the number of migrating cells in the si-CEBPB + SERPINA1 group was significantly higher than that in the si-CEBPB+Control group (Fig. 8C, D). Western blotting was used to detect various indicators of SERPINA1 and STAT3. The results showed that after knocking down CEBPB in RKO and Caco2 cells, the expression of SERPINA1, p-STAT3, and c-myc decreased. However, when SERPINA1 was overexpressed while knocking down CEBPB, the expression of p-STAT3 and c-myc increased (Fig. 8E). Our results indicated that the transcription factor CEBPB controls the transcription of SERPINA1 regulating the activation of the STAT3 pathway, thereby affecting CRC proliferation and migration (Fig. 8F).

## Discussion

Our study identified SERPINA1 as a colorectal cancer liver metastasis-related gene by analyzing the sequencing data. In a bioinformatic analysis of invasive ductal carcinoma, SERPINA1 was identified as a prognostically significant gene, participating in and establishing an effective prognostic model [23]. In a study related to thyroid cancer, SERPINA1 was found to be associated with lymph node metastasis of thyroid cancer and significantly associated with immune cell infiltration [24]. In earlier reports related to lung cancer, SERPINA1 expression was found to promote the proliferation and migration of lung cancer. High expression of SERPINA1 in plasma is associated with poor prognosis and advanced clinical stage of lung cancer [20]. We established stable colorectal cancer cell lines with SERPINA1 overexpression and knockdown. Through colony formation, EDU and MTS assays, we verified that SERPINA1 promoted the migration and invasion of colorectal cancer. In addition, through Transwell and wound healing assays, we confirmed that SERPINA1 promoted the migration and invasion of colorectal cancer. Using a liver metastasis model in nude mice by splenic injection, we also confirmed the pro-metastatic effects of SERPINA1.

The continuous activation of STAT3 can significantly increases c-myc and Survivin expression, thereby accelerating the growth of colon cancer cells [25]. It was found that Th17-type cytokines, IL-6 and TNF-α synergistically activate STAT3 to promote the growth of colorectal cancer cells [26]. SLCO1B3 can promote the proliferation and metastasis of colorectal cancer by activating the STAT3 pathway [27]. Previous studies have confirmed that the activation of STAT3 is related to the poor prognosis of tumors, including head and neck tumors, B-cell lymphoma, cervical cancer, gastric cancer, colon cancer, liver cancer, glioma and esophageal cancer [28]. STAT3 is also an important prognostic marker for colorectal cancer. In a study on colorectal cancer, Teppei Morikawa and others analyzed the p-STAT3 immunohistochemical staining results of 724 colorectal cancer pathologies and found that p-STAT3 was significantly associated with poor prognosis in colorectal cancer patients [29]. Furthermore, the activation of JAK1-STAT3-related signals plays a significant role in the metastasis of colorectal cancer [30]. These studies have all demonstrated the important role of the STAT3 pathway in the proliferation and migration of colorectal cancer. In addition, our study also found that overexpression of SERPINA1 can increase the expression of p-STAT3 in the nucleus. Phosphorylated STAT3 forms homodimers and enters the nucleus, allowing for signal exchange between the cytoplasm and nucleus. After entering the nucleus, p-STAT3 forms a complex with some auxiliary factors and binds to the promoter region of target genes to activate their transcription [31].

In addition, our study found that after increasing the expression of SERPINA1 in colorectal cancer cells, the expression of c-myc also increased significantly. C-myc is an important downstream effector of STAT3 signaling and is involved in the proliferation and migration of colorectal cancer cells [32]. A recent study on colorectal cancer showed that c-myc-activated LncRNA MNX1-AS1 promotes the proliferation of colorectal cancer by stabilizing YB1. In addition, SREBP1 interacts with c-myc to enhance the binding of c-myc to the SNAIL promoter of stromal genes, thereby increasing the expression of SNAIL and accelerating epithelial-mesenchymal transition, promoting tumor metastasis [33]. C-myc plays a key role in the proliferation of colorectal cancer, and high levels of c-myc expression are a negative prognostic marker for colorectal cancer and may become an important target for the treatment of CRC cancer [34]. In summary, we found that SERPINA1 can promote the proliferation, migration, and liver metastasis of colorectal cancer and plays a key role in the progression of colorectal cancer.

In a previous study, researchers found that the transcription factor C/EBP Homologous Protein (CHOP) cooperates with c-JUN to upregulate SERPINA1, thereby exacerbating the burden of proteotoxicity, which plays a significant role in liver disease progression [35]. In our study, transcription factors for SERPINA1 were predicted using the Cistrome and PROMO databases. CEBPB was selected for further validation based on its prognostic significance and expression correlation. Studies have shown that CEBPB plays an important role in tumors. In a study on gastric cancer, it was shown that DDIT3 can directly upregulate the transcription factor CEBPB, thereby increasing the stemness of gastric cancer cells and the expression of stem cell markers: SOX2, NANOG, OCT4, and CD133 [36]. At the same time, there are also studies reporting that CEBPB plays a pro-cancer role in bladder cancer [37]. CEBPB is involved in the transcriptional regulation of LncRNA UCA1, promoting its high expression and thereby promoting the proliferation of bladder cancer cells. KDM6B promotes the proliferation and migration of esophageal squamous cell carcinoma by increasing the transcriptional activity of CEBPB [38]. In a study on glioma, it was shown that CEBPB is associated with the expression of NQO1 and GSTP1 and can regulate the expression of antioxidant enzymes to regulate oxidative stress and mediate tumor growth in the brain [39]. In another study on breast cancer, upregulated CEBPB/AEP (asparaginyl endopeptidase) can mediate oxidative stress and promote lung metastasis of breast cancer [40]. However, the role of CEBPB in lung cancer is quite controversial, possibly because the function of CEBPB depends on the transcription factors cooperating in each cellular environment. In a study on non-small cell lung cancer, the drug resistance of NRF2-activated lung cancer was achieved through the cooperative function of NRF2 and CEBPB [41].

In our research used two cell lines (RKO and Caco2) to demonstrate that CEBPB has a positive transcriptional regulatory effect on SERPINA1 and regulates the proliferation and migration of CRC by activating STAT3 pathway. However, our study also has limitations. Despite analyzing the activation of the colorectal cancer JAK1-STAT3 pathway by SERPINA1 expression through multidimensional prediction and experimental validation, the mechanistic insights into how SERPINA1 upregulation results in JAK1 phosphorylation remain to be explored. We have planned to design and conduct a series of experiments aimed at elucidating the molecular pathways through which SERPINA1 affects JAK1 phosphorylation. This will involve the use of various techniques and methods, including co-immunoprecipitation and protein-protein interaction assays.

## Conclusion

In summary, our findings suggest that SERPINA1 is highly expressed in CRC and associated with tumor progression. Mechanistically, we revealed that SERPINA1 might mediate CEBPB-induced promotion of tumor cell growth and migration via STAT3 signaling for the first time. These results provide new clues to therapeutic target for CRC.

## Methods

### Tissue and cell lines

Fresh CRC samples (*n* = 17) and adjacent normal samples (*n* = 15) were collected form CRC patients without treatment from Liaoning Province Cancer Hospital sample library. All the specimens were frozen quickly in liquid nitrogen. Our research was approved by the Ethics Committee of Liaoning Province Cancer Hospital. Normal human intestinal epithelial cell (HIEC) was obtained from Otwo Biotech. Human CRC cell lines (RKO and Caco2) were obtained from Cell Bank of the Chinese Academy of Science.

RKO-H and Caco2-H: In the early stage of the project, the research group established a liver metastasis model by injecting RKO and Caco2 cells into nude mice through splenic injection (spleen preservation method). After 4 weeks, the mice were killed using cervical dislocation method, and the liver tissue was taken out and chopped. The tissue was washed with sterile PBS and centrifuged to remove the supernatant. After digestion with 0.05% trypsin and filtration with a nylon cell filter, it was centrifuged and the supernatant was discarded.

Subsequently, the cultured tumor cells were injected into nude mice through splenic injection. This process was repeated for a total of 4 rounds to obtain RKO-H and Caco2-H. HIEC was cultured using 1640 medium (BasalMedia) supplemented with 10% fetal bovine serum (FBS). HIEC was cultured using MEM (BasalMedia) supplemented with 10% FBS.

### Public datasets collection and preprocessing

We acquired independent CRC datasets GSE49355 [42], GSE110224 [43], GSE164191 [44], GSE10715 [45], GSE17537 [46], GSE12945 [47] form GEO database using R package GEOquery [48] and independent datasets. The data was normalized using normalizeBetweenArrays with R package limma [49]. The transcriptome profiles (FPKM) of primary colon adenocarcinoma and rectal adenocarcinoma were downloaded from TCGA database [50]. We merged these two datasets as TCGA-CRC dataset, which included 612 CRC patients.

### Differently expressed analysis

To further explore the mechanism in the change of metastatic ability of RKO-H and Caco2-H cells compared with RKO and Caco2 cells, we sent these four types of cells for transcriptome sequence. Based on the RNA-seq results, edgeR package was used to analyze differences between RKO-H and RKO, Caco2-H and Caco2 respectively [51]. The statistical threshold of Differently expressed genes (DEGs) was set as |log2FC| > 1 and *P* < 0.05. Meanwhile, we divided the GSE49355 dataset into colorectal liver metastasis (*n* = 19) and CRC primary tumors (*n* = 20). We then conducted a differential analysis between the two groups using the limma package, setting the threshold at | log2FC| > 1 and *P* < 0.05.

### Western

Lysate (Beyotime P0013J) containing protease inhibitor and EDTA was utilized to lyse tissues and cells. Protein was then separated by 10% PAGE gels (Epizyme PG112) and transferred onto PVDF membranes (Millipore IPVH00010). The PVDF membranes were blocked with 10% bovine serum albumin (BSA) and incubated with primary antibodies β-actin (CST 3700S), A1AT (Proteintech 16382-1-AP), c-myc (SANTA sc-40), lamin B1 (CST13435S), p-JAK1 (CST 3331S), JAK1 (CST 3332S), p-STAT3 (CST 9145S), STAT3 (CST 4904S) and CEBPB (Proteintech 23431-1-AP) at 4 °C overnight. Then, the membranes were incubated with secondary antibody (Zsbio zb-2301 and Zsbio zb-2305) at room temperature for 1 h.

### RTq-PCR (real-time quantitative PCR)

We used TRIzol reagent (Invitrogen) to lyse tissues and cells to extract total RNA. Genomic DNA removal and reverse transcription were performed by primeScript RT Reagent Kit (TaKaRa). RTq-PCR was implemented using TB Green Premix Ex TaqII (TaKaRa), Primer, Sterilized distilled water and cDNA. The primers used were as follows: GAPDH F: CTCTGCTCCTCCTGTTCGAC, R: GCGCCCAATACGACCAAATC; SERPINA1 F: GATCAACGATTACGTGGAGAAGG, R: CCTAAACGCTTCATCATAGGCA; CEBPB F: CTTCAGCCCGTACCTGGAG, R: GGAGAGGAAGTCGTGGTGC.

### Transwell

Transwell inserts (Corning #3422) were used to perform Transwell migration and invasion experiments. CRC cells (1*10^5^) were seeded in the upper chambers in serum-free MEM and 20% FBS MEM was added to the lower chamber. Following a 36-hour incubation period at 37 °C, the cells that had migrated to the underside of the upper chambers were fixed using methanol, stained with crystal violet, and imaged under a microscope. The experiment steps of Transwell invasion experiment were as same as those of Transwell migration, except that 75 microliters of extracellular matrix gel were added to the upper chamber (Corning #456234).

### Wound-healing assay

We used a 6-well plate and drew horizontal lines on the backside of each hole. Every 6 × 10^5^ cells per well was seeded into each well. Then, a scratch was made on the cells utilizing a 10 μL pipette tip in a direction perpendicular to the horizontal line. Tumor cells were incubated with serum-free medium at 37 °C and imaged at 0, 18 and 36 h.

### Colony formation

RKO, RKO-H, Caco2 and Caco2-H were digested with trypsin and resuspended to form single-cell suspension. Then, 800 cells per well were placed in a six-well plate and incubated at 37 °C. After washing with PBS once, the cells were fixed with methanol for 20 min. After staining with 1% crystal violet for 20 min, the excess dye was washed away with PBS and the cells were left to dry before image acquisition.

### MTS and EDU

MTS assay (3-(4,5-Dimethylthiazol-2-yl)-5-(3-carboxymethoxyphenyl)-2-(4-sulfophenyl)-2H-tetrazolium assay) is a commonly used experimental method for evaluating cell growth and proliferation ability. CRC cells (2.5 × 10^3^ for RKO-H/Caco2-H and 3 × 10^3^ for RKO/Caco2) were seeded in 96-well plates and cultured for different time periods (day 1, day 2, and day 3) at 37 °C. Prior to detection, 20 μL MTS reagent (Promega G3580) was added to each well. EDU (5-ethynyl-2-deoxyuridine) experiment is an effective method to evaluate cell proliferation. In our study, EDU experiment was performed with EDU cell proliferation detection kit (RiboBio C10310-3).

### Enrichment analysis

To investigate pathways associated with SERPINA1, TCGA-CRC patients were divided into low-SERPINA1 and high-SERPINA1 groups according to the median of SERPINA1 expression. R package GSVA to calculate the scores of related pathways for each sample [52, 53]. GSEA is an algorithm that analyzes whether there is a difference between two groups for a certain gene set [54]. Based on the c2.cp.kegg gene set from MSigDB, we calculated and displayed the pathways in the high-SERPINA1 group. We further calculated scores of each CRC sample with the ssGSEA algorithm, based on “KEGG JAK STAT SIGNALING PATHWAY” and “ST STAT3 PATHWAY” gene sets. Correlation between the scores of CRC and the expression level of SERPINA1 was analyzed by Spearman algorithm.

### Chromatin immunoprecipitation (ChIP)

ChIP assay was performed with ChIP kit (BersinBio). In brief, 2 × 10^7^ RKO cells were collected, cross-linked with 1% formaldehyde, extracted nucleus and sonicated. Then, lysates were incubated at 4 °C for 12 h with a specific CEBEP immunoprecipitation and IgG antibody. RTq-PCR was implemented to verify the binding of CEBPB to the *SERPINA1* promoter region. The primers specific for the *SERPINA1* promoter region included Site1 (ACTTGCAAAAC) primer F: CCCCACTTAGACCCCTGAGA, Site1 primer R: GCTCTGTTAGCATAATGGACACTTG; Site2 (GGTTTCCTCAT) primer F: CCAGCATCGCCTCATCTTAG, Site2 primer R: TGGGTCTCAGGGGTCTAAGTG.

### Dual-luciferase reporter assay

We acquired the 2000 bp and 1000 bp promoter sequences the *SERPINA1* from NCBI Gene database [55]. The luciferase vectors (pPRO-RB-Report) inserted with the two sequences were constructed by RiboBio. We performed with RKO and 293 T cell lines. The pPRO-RB-Report and si-CEBPB (Genepharma) were co-transfected into the cells by Lipo8000 (Beyotime). After 24 h, luciferase activity was detected with a dual luciferase reporter assay kit (Vazyme DL101-01).

### In vivo experiment

Four-week-old BALB/c nude mice were observed for 7 days in the laboratory before conducting the experiment. On the day of the experiment, 5 tubes containing 50 μL PBS with 2*10^6^ RKO-Control and 5 tubes containing 50 μL PBS with 2*10^6^ RKO-SERPINA1 were prepared respectively.

We utilized a computer-generated randomization to determine how animals were allocated to experimental groups. The sample size was carefully chosen based on former related studies and power analysis. Each nude mouse was injected with 1% pentobarbital sodium anesthesia into the abdominal cavity. After the mice were successfully anesthetized, they were fixed on the operating table and prepared for surgery. The skin of the mice was disinfected with iodine, and 0.5 cm incision was made along the left axillary line and rib margin. The spleen was pulled out from the abdominal cavity, and PBS containing tumor cells was injected into the mice. The needle hole was pressed with an alcohol cotton swab, and the abdomen was sutured. The mice were returned to their cages to continue feeding for 20 days, and then euthanized by cervical dislocation. The liver was isolated and photographed. The transfer area of the liver was measured using ImageJ software and statistically analyzed after measurement. In the whole process, we were blinded to the group allocation of mice.

### Immunohistochemistry

We used immunohistochemistry to detect A1AT expression in CRC tissues and analyzed the relationship between A1AT and clinicopathological stage. A1AT primary antibody (Abcam ab9399) and secondary antibody (Zsbio zb-2305) were used in the procedure. IHC score was evaluated based on the product of the percentage of stained positive cells and staining intensity.

### Statistical analysis

Throughout the experimental procedures and during the assessment of outcomes, the researchers were blinded to the group allocation of samples. Statistical analyses and graphical visualization were performed using R software (4.1.3). For the comparison between two continuous variables: we used Student’s *t*-test to assess the significance of differences between normally distributed variables, and the Wilcoxon rank-sum test to assess the significance of differences between non-normal distributed variables. Tests were performed to assess homogeneity of variance. The sample size was carefully chosen based on a power analysis to ensure adequate statistical power to detect the pre-specified effect size. The R package survminer was utilized to determine the optimal cutoff of SERPINA1 in KM plots. *P* < 0.05 was considered statistically significant.

### Supplementary information

Supplementary material legends

WB Raw data

Figure S1

Figure S2

Figure S3

## Data Availability

All data needed were present in the paper and/or the Supplementary material.
