# Supplementary material for: CEBPB-mediated upregulation of SERPINA1 promotes colorectal cancer progression by enhancing STAT3 signaling
Source: Cell Death Discov. 2024 May 6;10:219. doi: 10.1038/s41420-024-01990-9 (PMC11074302; doi:10.1038/s41420-024-01990-9)
Supplement: Supplementary file 1 — Supplementary material legends [file 41420_2024_1990_MOESM1_ESM.docx]

Figure S1: RKO-H and Caco2-H have higher proliferation and migration capabilities than parental RKO and Caco2.

A: Schematic diagram of RKO-H and Caco2-H cells. B: Colony formation assay of RKO and RKO-H. C: Colony formation assay of Caco2 and Caco2-H. D: Transwell migration assay of RKO and RKO-H. E: Transwell migration assay of Caco2 and Caco2-H. F: Wound healing assay of RKO and RKO-H. G: Wound healing assay of Caco2 and Caco2-H. Scale bar: 100μm. Error bars: standard deviations. * : *P* < 0.05, ** : *P* < 0.01, *** : *P* < 0.001.

Figure S2: Identification of potential colorectal cancer metastasis-related gene SERPINA1.

A-B: Volcano maps show DEGs of RKO-H and RKO, Caco2-H and Caco2. C: Heatmaps of top 50 DEGs of RKO-H and RKO, Caco2-H and Caco2. D: Volcano map shows DEGs of colorectal primary tumors and liver metastasis based on GSE49355. E: The Venn diagram displays the intersection of three groups, suggesting that SERPINA1 is a potential metastasis-related gene.

Figure S3: Clinical pathological stage analyses of FOXA1 and CEBPB based on TCGA-CRC.

A-D: Analysis of the clinical relevance of FOXA1. E-H: Analysis of the clinical relevance of CEBPB. NS: not significant, * : *P* < 0.05, ** : *P* < 0.01, *** : *P* < 0.001.
