# Supplementary material for: CEBPB-mediated upregulation of SERPINA1 promotes colorectal cancer progression by enhancing STAT3 signaling
Source: Cell Death Discov. 2024 May 6;10:219. doi: 10.1038/s41420-024-01990-9 (PMC11074302; doi:10.1038/s41420-024-01990-9)
Supplement: Supplementary file 2 — WB Raw data [file 41420_2024_1990_MOESM2_ESM.pptx]

## Slide 1
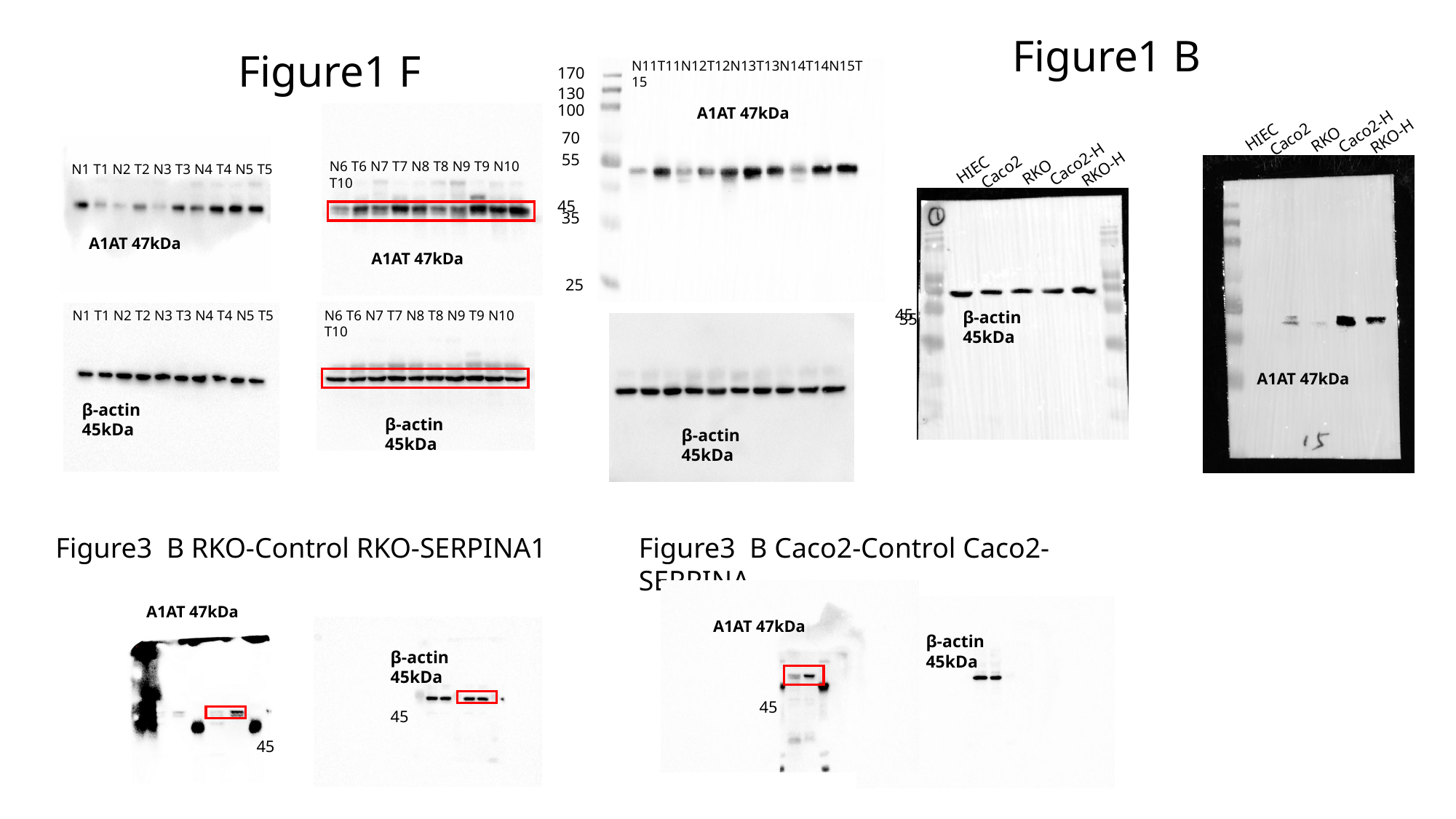

Figure1 B
Figure1 F
N11T11N12T12N13T13N14T14N15T15
170
130
100
A1AT 47kDa
Caco2-H
RKO-H
HIEC
 70
RKO
Caco2
 55
Caco2-H
N6 T6 N7 T7 N8 T8 N9 T9 N10 T10
RKO-H
HIEC
N1 T1 N2 T2 N3 T3 N4 T4 N5 T5
RKO
Caco2
 45
 35
A1AT 47kDa
A1AT 47kDa
 25
 45
N1 T1 N2 T2 N3 T3 N4 T4 N5 T5
N6 T6 N7 T7 N8 T8 N9 T9 N10 T10
β-actin 45kDa
 35
A1AT 47kDa
β-actin 45kDa
β-actin 45kDa
β-actin 45kDa
Figure3 B RKO-Control RKO-SERPINA1
Figure3 B Caco2-Control Caco2-SERPINA
A1AT 47kDa
A1AT 47kDa
β-actin 45kDa
β-actin 45kDa
 45
 45
 45

## Slide 2
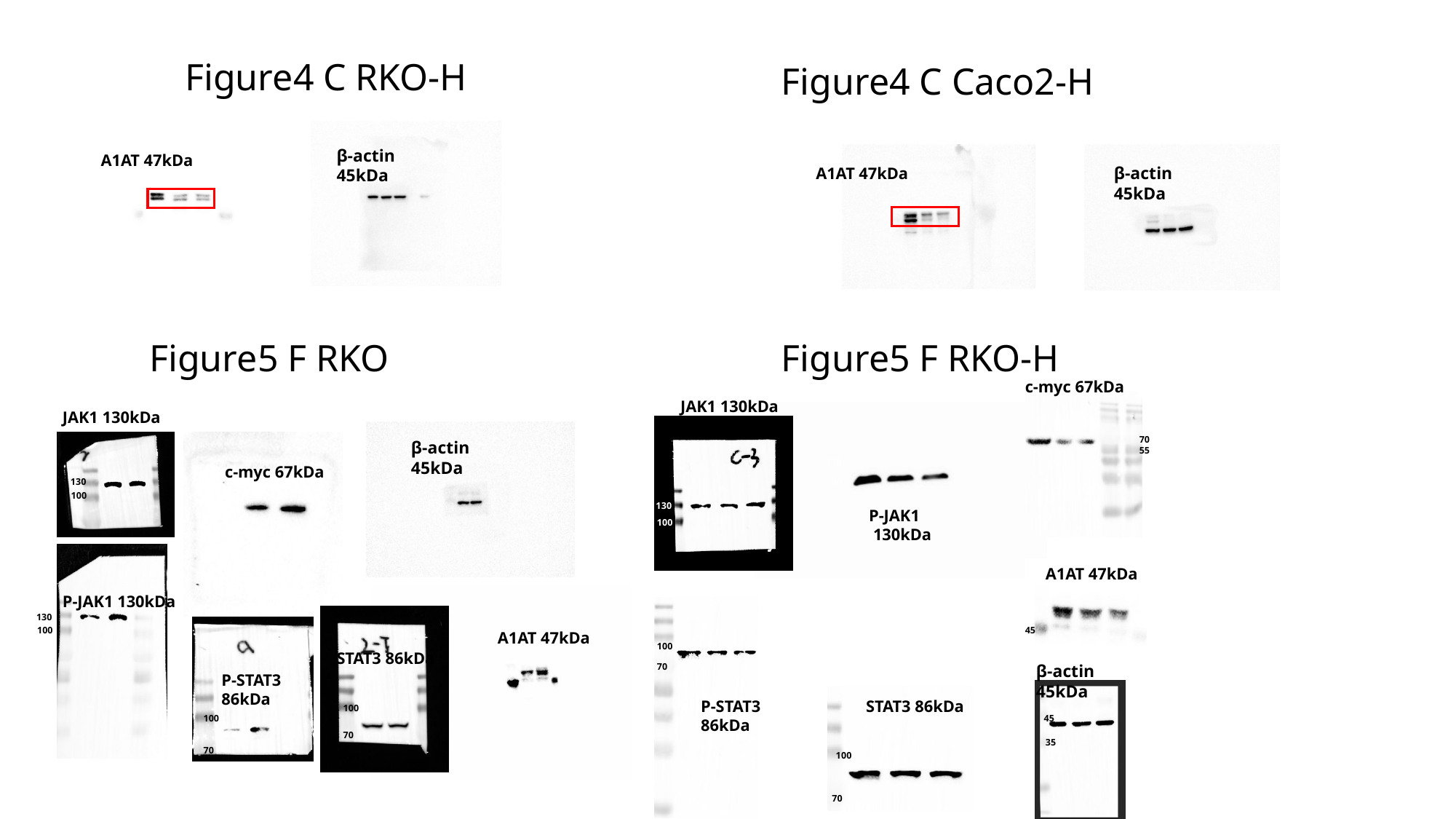

Figure4 C RKO-H
Figure4 C Caco2-H
β-actin 45kDa
A1AT 47kDa
β-actin 45kDa
A1AT 47kDa
Figure5 F RKO
Figure5 F RKO-H
c-myc 67kDa
JAK1 130kDa
JAK1 130kDa
70
β-actin 45kDa
55
 130
c-myc 67kDa
 130
100
100
 130
 P-JAK1
 130kDa
100
A1AT 47kDa
P-JAK1 130kDa
 130
100
45
A1AT 47kDa
100
STAT3 86kDa
70
β-actin 45kDa
P-STAT3 86kDa
P-STAT3 86kDa
STAT3 86kDa
100
100
45
70
35
70
100
70

## Slide 3
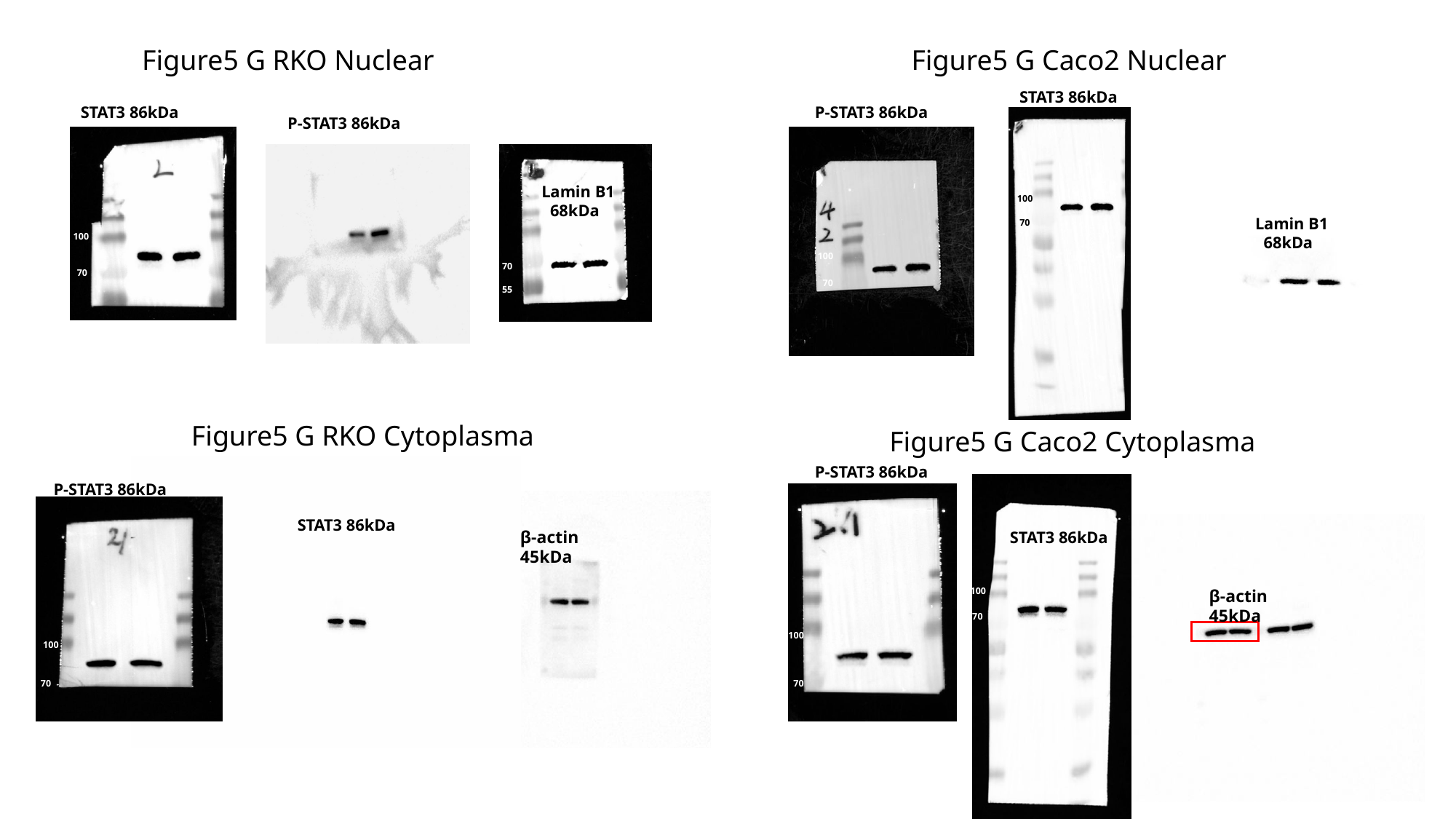

Figure5 G RKO Nuclear
Figure5 G Caco2 Nuclear
STAT3 86kDa
STAT3 86kDa
P-STAT3 86kDa
P-STAT3 86kDa
Lamin B1
 68kDa
 100
Lamin B1
 68kDa
70
 100
 100
70
70
70
55
Figure5 G RKO Cytoplasma
Figure5 G Caco2 Cytoplasma
P-STAT3 86kDa
P-STAT3 86kDa
STAT3 86kDa
β-actin 45kDa
STAT3 86kDa
 100
β-actin 45kDa
70
 100
 100
70
70

## Slide 4
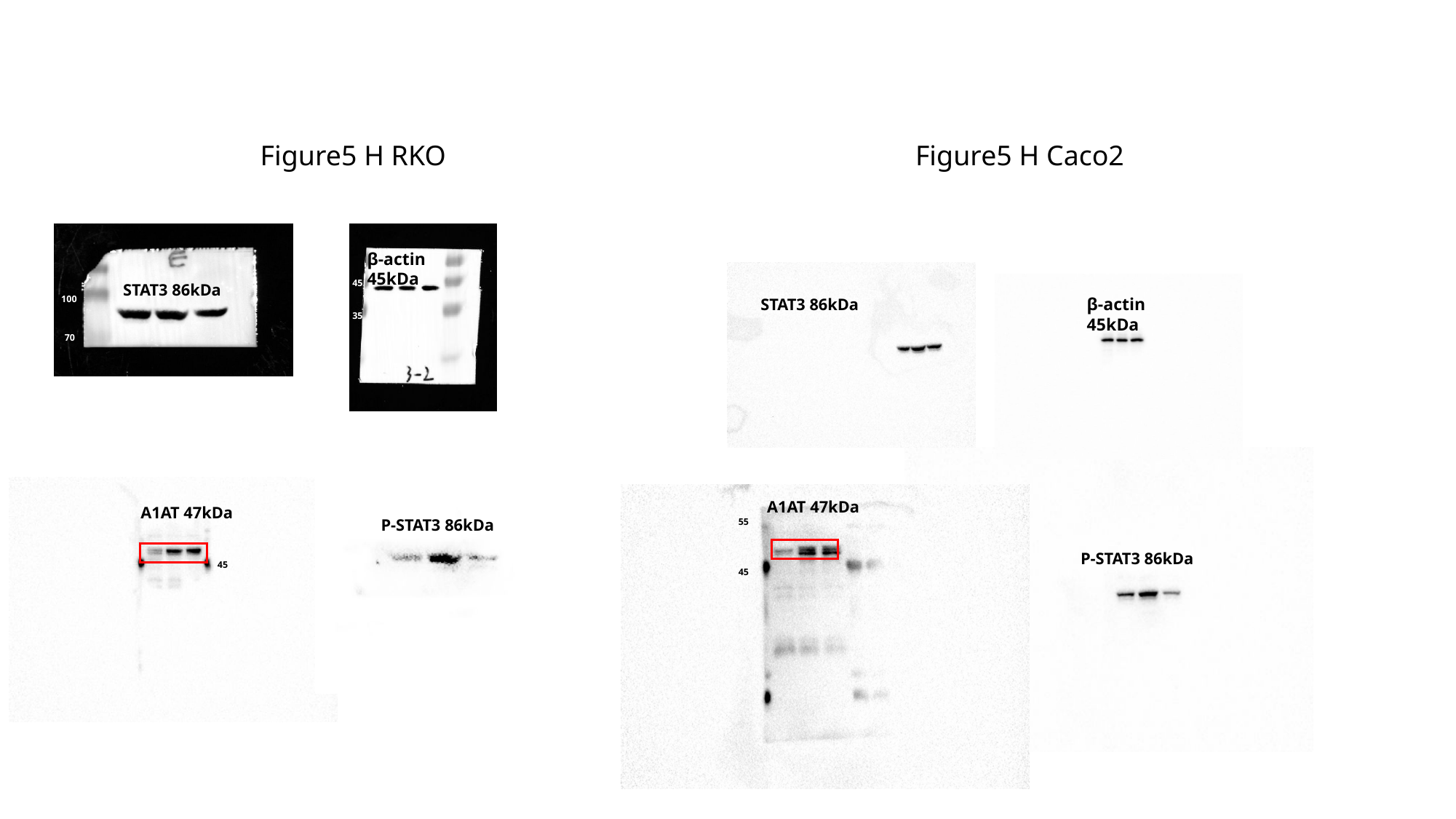

Figure5 H RKO
Figure5 H Caco2
β-actin 45kDa
45
STAT3 86kDa
45
 100
β-actin 45kDa
STAT3 86kDa
35
70
A1AT 47kDa
A1AT 47kDa
P-STAT3 86kDa
55
P-STAT3 86kDa
45
45

## Slide 5
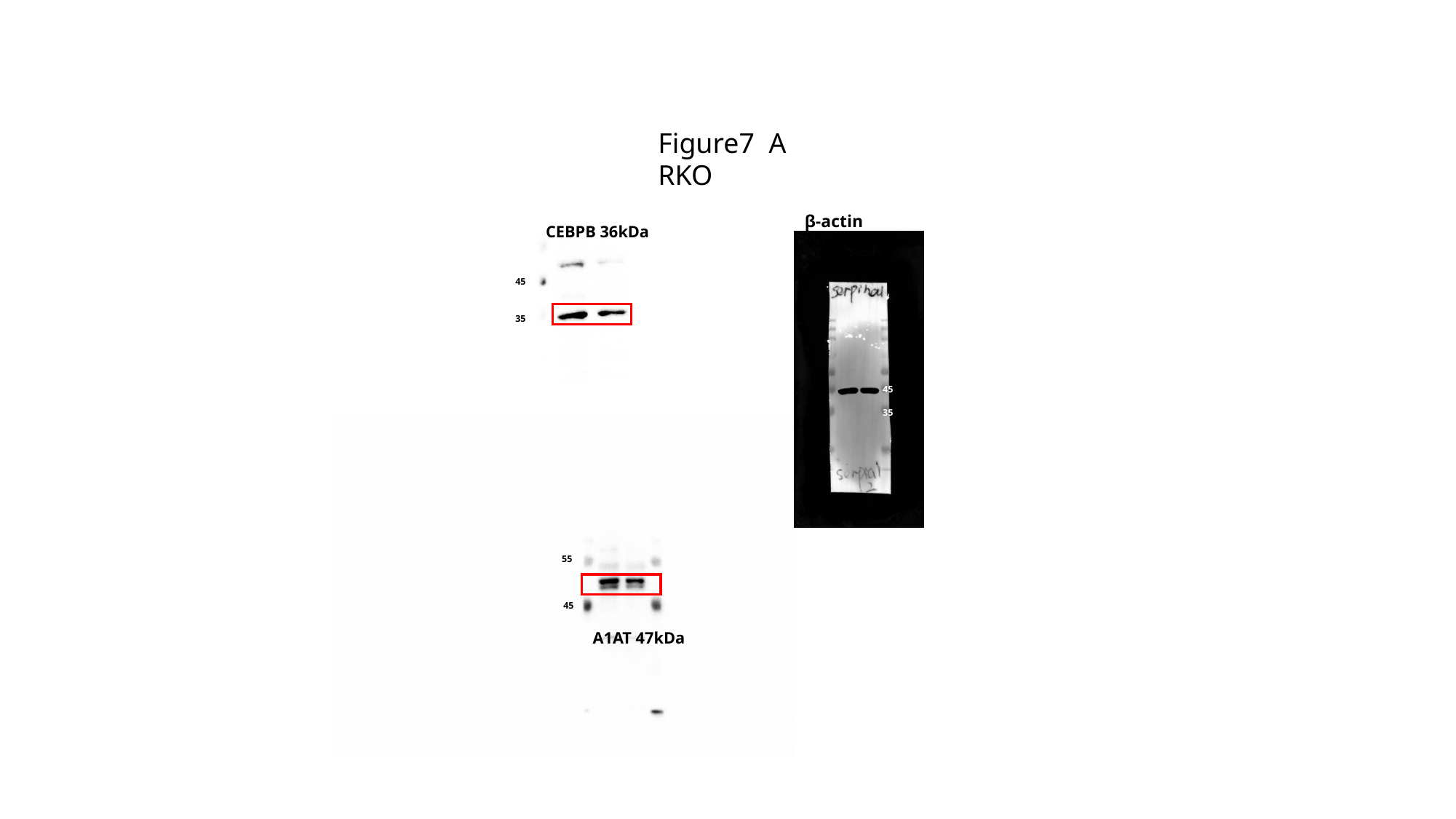

Figure7 A RKO
β-actin 45kDa
CEBPB 36kDa
45
35
45
35
55
45
A1AT 47kDa

## Slide 6
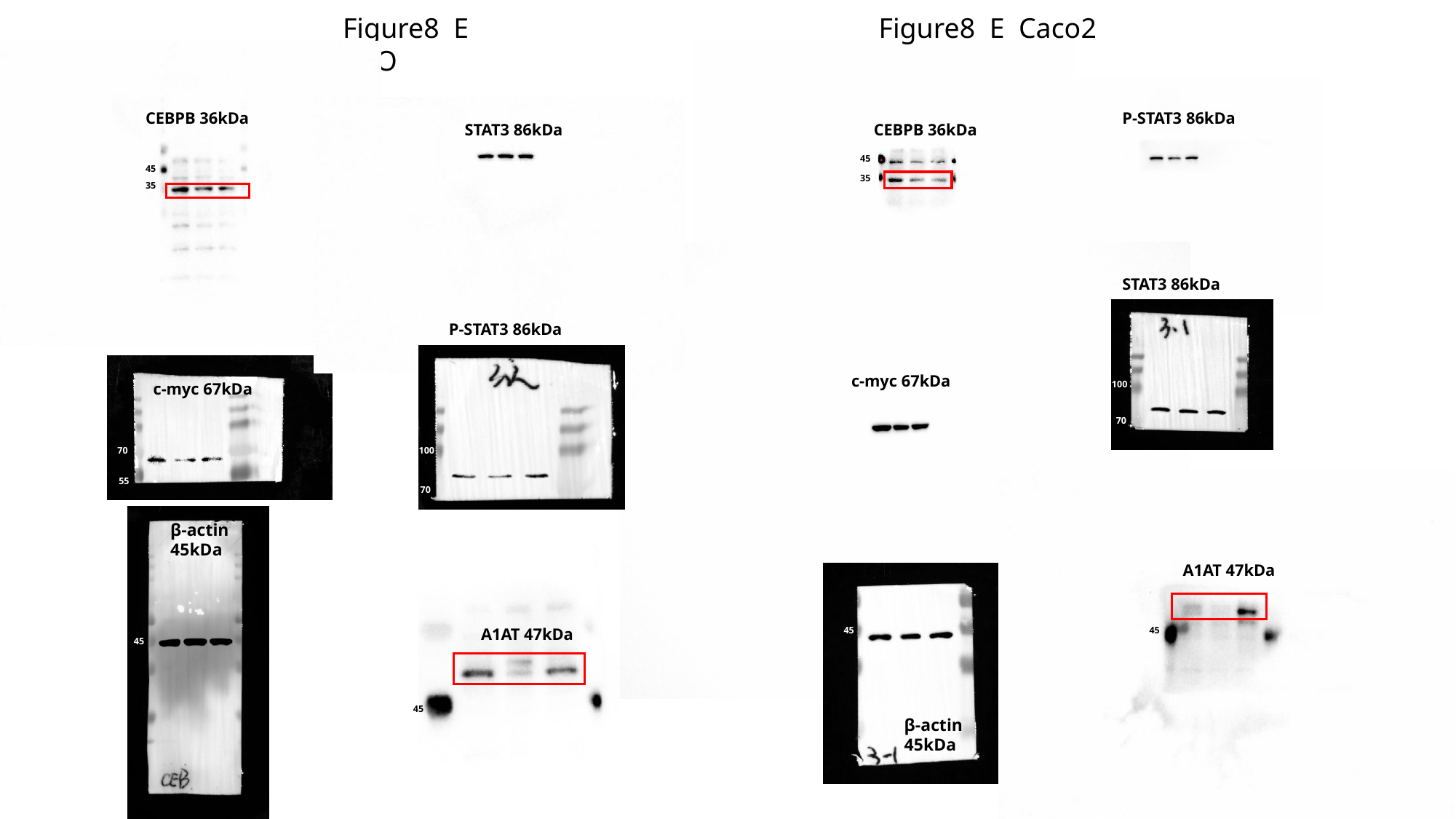

Figure8 E RKO
Figure8 E Caco2
CEBPB 36kDa
P-STAT3 86kDa
STAT3 86kDa
CEBPB 36kDa
45
45
35
35
STAT3 86kDa
P-STAT3 86kDa
c-myc 67kDa
c-myc 67kDa
100
70
70
100
55
70
β-actin
45kDa
A1AT 47kDa
A1AT 47kDa
45
45
45
45
β-actin
45kDa
