# Supplementary figures and images for: CEBPB-mediated upregulation of SERPINA1 promotes colorectal cancer progression by enhancing STAT3 signaling
Source: Cell Death Discov. 2024 May 6;10:219. doi: 10.1038/s41420-024-01990-9 (PMC11074302; doi:10.1038/s41420-024-01990-9)

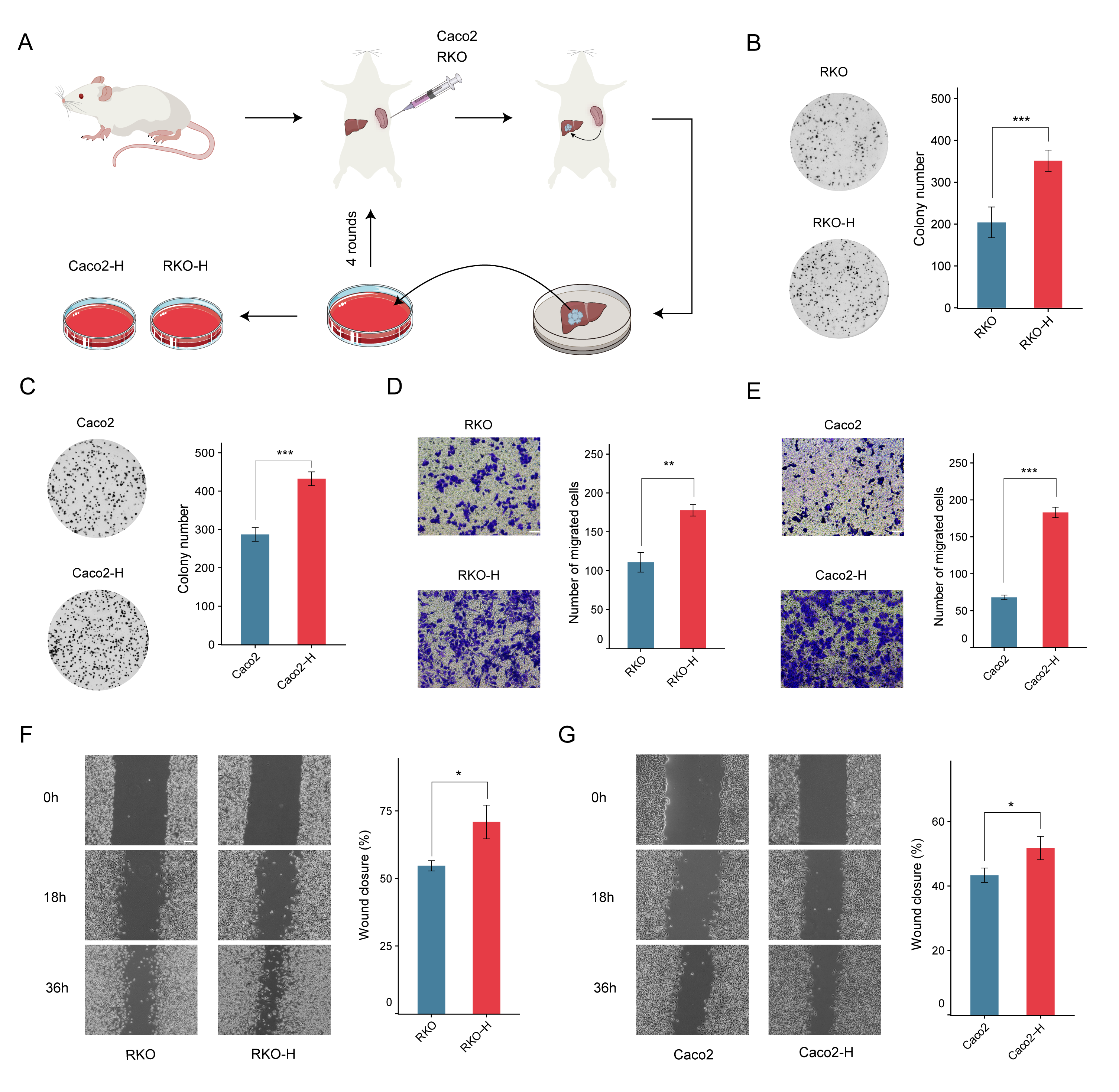

Supplement: Supplementary file 3 — Figure S1 [file 41420_2024_1990_MOESM3_ESM.tif]

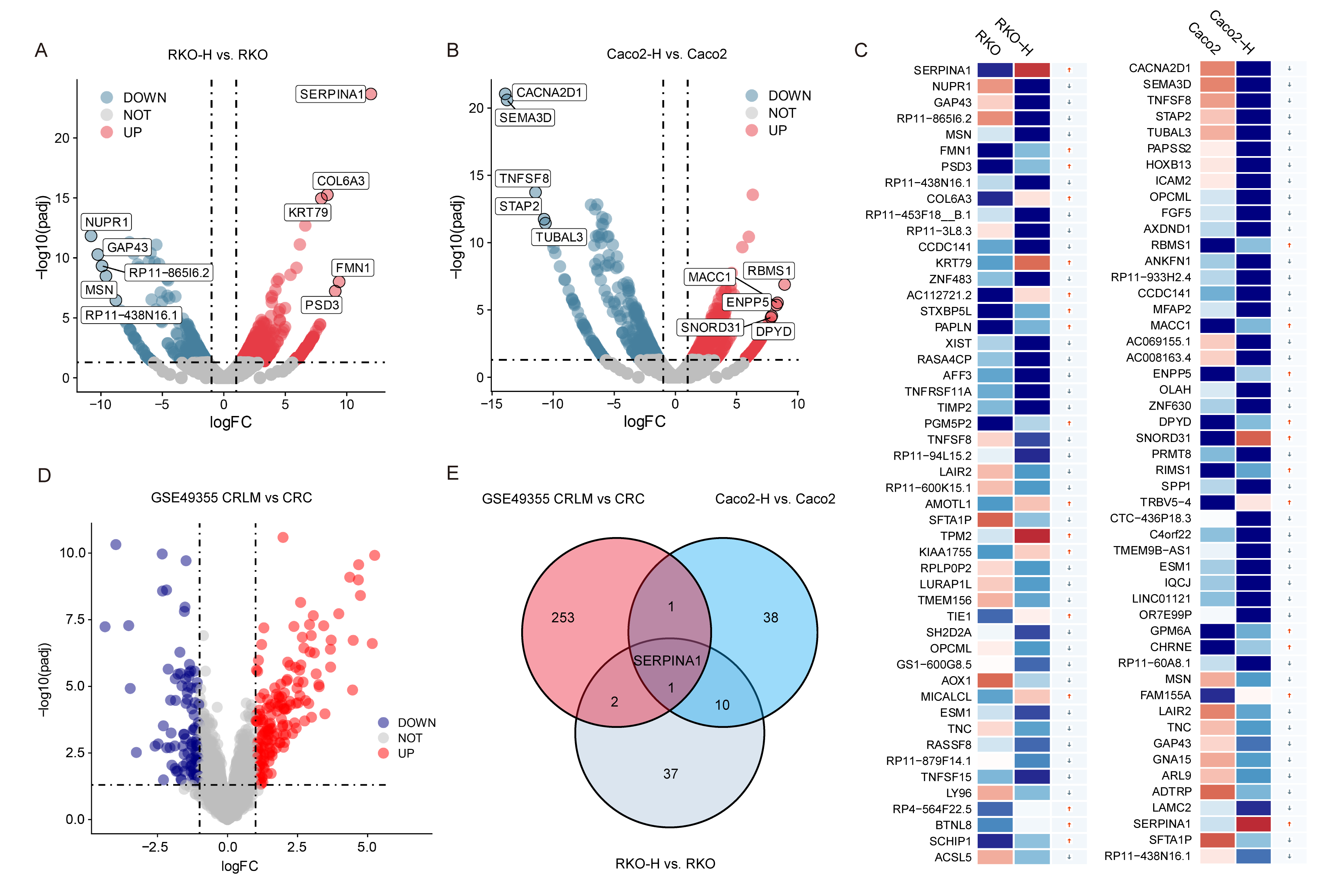

Supplement: Supplementary file 4 — Figure S2 [file 41420_2024_1990_MOESM4_ESM.tif]

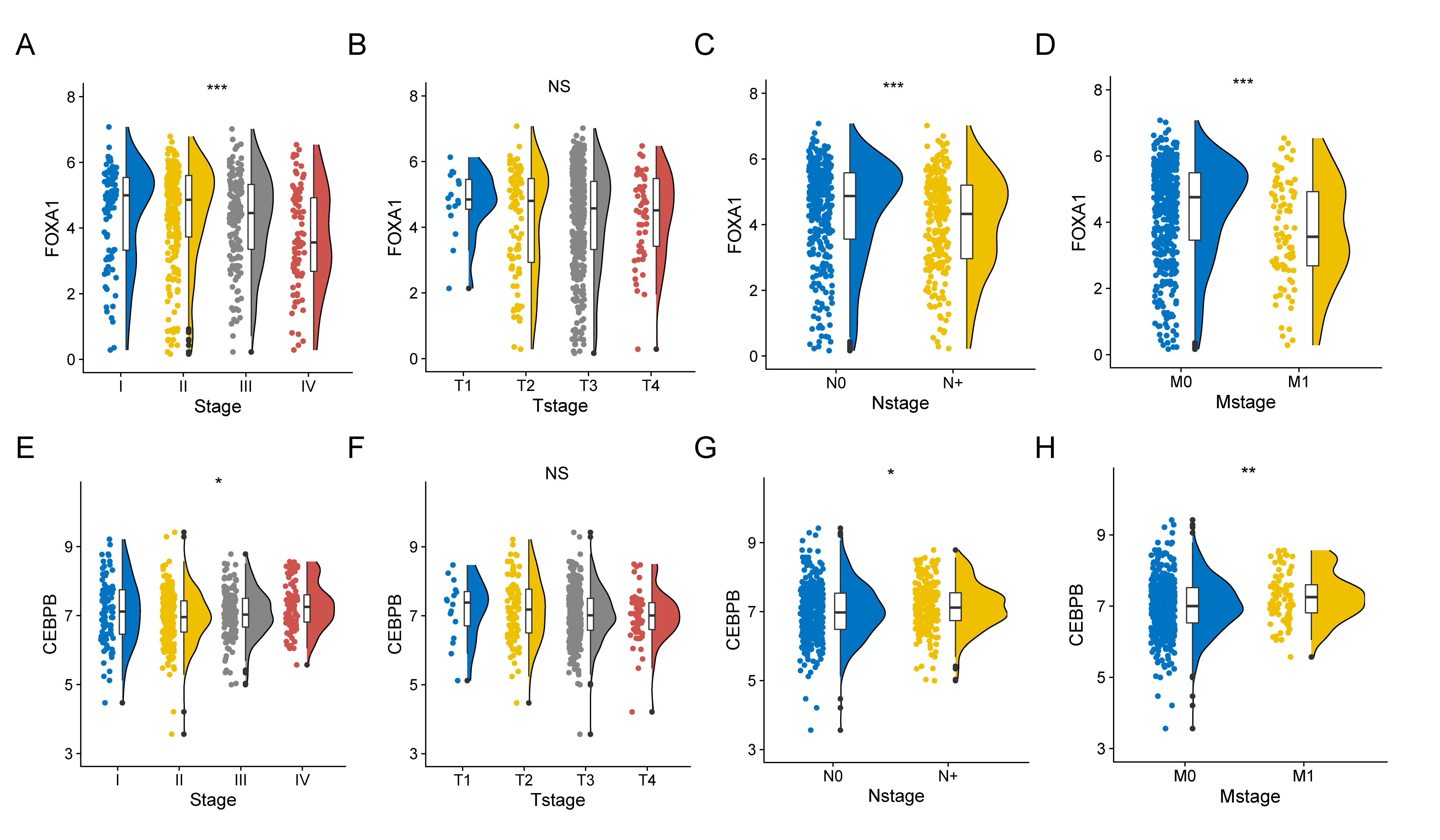

Supplement: Supplementary file 5 — Figure S3 [file 41420_2024_1990_MOESM5_ESM.tif]
